# Supplementary material for: Content-rich biological network constructed by mining PubMed abstracts
Source: BMC Bioinformatics. 2004 Oct 8;5:147. doi: 10.1186/1471-2105-5-147 (PMC528731; doi:10.1186/1471-2105-5-147)
Supplement: Additional File 2 — The original results of the above study (non-essential files are deleted to keep the file size under the limit set by BMC bioinformatics). [file 1471-2105-5-147-S2.bz2 › chilibotAdditionalFile2/dip05/726ID9822594E18075/html/HLYD_HLYA.html]

 


 **HLYD** and **HLYA** 
  
Found 65 abstracts in PubMed, retrieved 05.  
 

 What does Google say? 
 PDF only 
| .edu only 

---

**Interactive relationship** (e.g. stimulation, inhibition, etc)

**Neutral relationship**- Our data suggest that TnhlyAs generated hemolysin  [ **HLYA** ]  fusion proteins are secreted efficiently by the HlyB  **HlyD**  TolC hemolysin  [ **HLYA** ]  secretion machinery.  Ref: 9711856 FEMS Microbiol Lett, 1998

**Non-interactive relationship** (e.g. studied together, co-existance, homology, etc.)

- The structure and function of the components of the  **HlyA**  secretion apparatus, HlyB,  **HlyD**  and TolC, have been studied in great detail.  Ref: 11755084 Trends Microbiol, 2002
- The toxin  **HlyA**  is exported from Escherichia coli, without a periplasmic intermediate, by a type I system comprising an energized inner membrane IM translocase of two proteins,  **HlyD**  and the traffic ATPase HlyB, and the outer membrane OM porin like TolC.  Ref: 9822594 EMBO J, 1998
- We show that an scFv  **HlyA**  hybrid secreted by Hly type I transporter TolC, HlyB,  **HlyD**  is accumulated in the extracellular medium with the disulphide bonds correctly formed.  Ref: 11309117 Mol Microbiol, 2001
